# An interview with Dan L. Longo, Section Editor of the Malignant hematological diseases section

**DOI:** 10.1186/2052-1839-13-2

**Published:** 2013-04-10

**Authors:** Dan L Longo

**Affiliations:** 1 Hematology Division, Department of Medicine, Brigham and Women’s Hospital, 10 Shattuck St, Boston, MA, 02115, USA

## Abstract

Dan L. Longo is professor of medicine at Harvard Medical School in the division of hematology at Brigham and Women’s Hospital and Deputy Editor of the *New England Journal of Medicine*. He is a Master of the American College of Physicians and is a member of the American Society of Clinical Investigation and the Association of American Physicians. He has been active in research on the treatment of malignant lymphoma including new treatments targeting CD30, CD40, HLA Class II molecules and immunoglobulin idiotype. His laboratory work has focused in part on the regulation of lymphocyte proliferation, NK cell effects on hematopoiesis, and tumor-induced immunosuppression. He is the Section Editor of the Malignant hematological diseases section for *BMC Hematology*. In this interview, he explains how he first became interested in hematology and gives his personal view on the recent progress and future challenges of the hematological cancer field in particular.

## How did you first become interested in hematology?

I was exposed to outstanding hematologists in medical school who were also great teachers, Patrick Henry, Charles Mengel, and Victor Herbert. I found the subject interesting and the patients fascinating. Looking at peripheral blood smears and bone marrow specimens was aesthetically pleasing as well as intellectually challenging. I had the opportunity to work in Dr. Herbert’s laboratory during my senior year and his amazing depth and breadth of knowledge and innovative research ideas got me hooked on the notion of an academic career in hematology.

## How did you end up focusing on malignant hematological diseases?

After my training in internal medicine at the Brigham in Boston, I went to the National Cancer Institute where Vincent DeVita, Robert Young, and Bruce Chabner played a major role in teaching me about cancer in general and hematologic malignancies in particular. It was the mid-1970’s and the curability of lymphomas with chemotherapy had just been established and knowledge of immunology was beginning to burgeon. The amazing diversity of lymphocyte phenotypes was becoming apparent and the notion of lymphomas as tumors of specific types of lymphocytes began to emerge from work from Lee Nadler, Sam Broder, and others. I was fortunate to collaborate with Stan Korsmeyer at NCI in his studies on bcl2 in follicular lymphoma. Hybridoma technology made it possible to develop an almost unlimited supply of targeted therapies. It seemed that empiric treatment success was being complemented by increasing biologic insights and new treatment approaches were being created—new drugs, monoclonal antibodies, immune therapies. It felt like we were at the beginning of a revolution in lymphoma treatment. And advances in lymphoma treatment had led the way to advances in treating other types of cancers. I wanted to be a part of that expansion of the therapeutic armamentarium based on the rational selection of targets.

## Why is it an exciting time to be involved with hematological cancer research in particular?

Remarkable progress is being made in elucidating molecular mechanisms involved in tumor biology and defining tumor cell vulnerabilities. Many new therapies are showing promising antitumor effects: proteasome inhibitors and lenalidomide in myeloma, arsenic and transretinoic acid in promyelocytic leukemia, alk inhibitors in anaplastic large cell lymphoma, BRAF inhibitors in hairy cell leukemia, new third generation abl kinase inhibitors in chronic myeloid leukemia, Bruton’s tyrosine kinase inhibitors in chronic lymphoid leukemia, adoptive transfer of lymphocytes with chimeric antigen receptors engineered to recognize tumors of various types, and this list is not complete. Genetic lesions in virtually every form of hematologic malignancy have been identified and although much more knowledge needs to be developed, rational therapeutic targets have not all been explored. New ways of interfering with driver mutations and new ways of replacing essential functions damaged by mutation in tumor cells are being assessed. For the foreseeable future, research on hematologic cancer will be leading to new therapies and combinations of therapies to further improve survival.

## What do you think are the future directions of research into this field?

We cannot let the partial success of novel interventions targeting abnormal enzymes blind us to the prior successes achieved by chemotherapeutic agents. I think the future will involve several areas for progress: continued definition of molecular targets for intervention, better definition of prognostic subsets of patients based on molecular features, use of the newer targeting agents in combination with each other and with cancer chemotherapeutic agents to achieve even better long-term survival and cure, elucidation of host-tumor interactions and their alteration to boost host defenses, immunologic and vaccine approaches to treatment and prevention, increased attention to late effects of treatment and their prevention.

## What challenges and developments can we expect to see in the next few years?

A major challenge is the requirement for national and international collaboration to take the next steps. Many hematologic malignancies have quite high rates of response and control with existing therapies. Studies to show an improvement from 60% to 80% long-term survival require more subjects than studies to show an improvement from 15% to 35% long-term survival. Furthermore, the many (and increasing) treatment choices complicate study design. The numbers of patients required for meaningful clinical trials and the abundant ideas for novel therapies and their integration into standard treatments represent an enormous challenge to the field. It is my hope that researchers can come together to work collaboratively to take the treatment of hematologic malignancies to next level of success.

## What does the future hold for the field of hematological cancer and what do you think are its limitations?

It is my expectation that the field will continue to make substantial improvements in the treatment of patients with hematologic malignancies. It is expected that there will be setbacks. Tumors adapt. Resistance may develop. But it is hoped that the insights that emerge from studying resistance will provide clues to overcoming it in the next generation of treatments. We must also keep in mind the remarkable heterogeneity in tumor cells. A fundamental insight in cancer 50 years ago was its clonal origins. However, until recently, the wide array of genotypes that evolve in a tumor over time was not fully appreciated. We will have to be careful to choose therapeutic targets that are likely shared by all the tumor cells rather than alterations in clonal variants that account for a small fraction of the tumor.

## Are there any particular papers you would like to see submitted to your section?

We hope the journal becomes a venue for interesting new findings including relevant basic science insights and clinical applications. Well-done negative clinical trials may also help us take the next step and should be considered if the result is an important addition to the knowledge base.

## Competing interests

Dan L. Longo is a Section Editor for *BMC Hematology*.

## Pre-publication history

The pre-publication history for this paper can be accessed here:

http://www.biomedcentral.com/2052-1839/13/2/prepub

